# Safety and efficacy of VisuMax® circle patterns for flap creation and enhancement following small incision lenticule extraction

**DOI:** 10.1186/s40662-015-0031-5

**Published:** 2015-12-26

**Authors:** Ekktet Chansue, Morakot Tanehsakdi, Sukanda Swasdibutra, Colm McAlinden

**Affiliations:** TRSC International LASIK Center, 6th Floor, U Chu Liang Boulevard, 968 Rama 4 Road, Bangkok, Thailand; Flinders University, Bedford Park, Adelaide, South Australia Australia; Wenzhou Medical College, Wenzhou, Zhejiang China

**Keywords:** Small incision lenticule extraction, SMILE, Lenticule, Residual lenticule extraction, Flap creation, Circle pattern, Refractive enhancement, Femtosecond laser, Refractive surgery

## Abstract

**Background:**

The purpose of this case series is to evaluate the safety and efficacy of VisuMax® Circle patterns in eyes that have undergone small incision lenticule extraction, thus creating a flap to perform an enhancement procedure or residual lenticule extraction.

**Methods:**

This prospective, single center, case study series evaluated the use of a VisuMax® Circle pattern to create a corneal flap following small incision lenticule extraction. Patients were treated and followed at TRSC International LASIK Center (Bangkok, Thailand) for 3 months to assess the efficacy and safety of the procedure. Efficacy was determined by the surgeon’s ability to lift the created corneal flap.

**Results:**

The study enrolled 28 eyes. Twenty-seven underwent the VisuMax® Circle pattern procedure for refractive enhancement, and one for residual lenticule extraction. In 100 % of cases (28 eyes) the lifting of the flap was possible, as planned. In all cases of refractive enhancement (27 eyes) by laser in situ keratomileusis (LASIK), the exposure of the stromal bed was sufficient for the necessary excimer laser ablation. No eyes lost two or more Snellen lines of corrected distance visual acuity (CDVA) and no procedure or flap-related complications or serious adverse events occurred.

**Conclusions:**

This initial case series demonstrates that VisuMax® Circle pattern is efficacious and a suitable method to create a corneal flap for enhancement, following small incision lenticule extraction.

## Background

New technologies such as femtosecond lasers are creating a paradigm shift in the surgeon’s ability to perform refractive correction with improved results [1–5]. The development of small incision lenticule extraction (SMILE) as a new, flapless procedure has been a major innovation in corneal refractive surgery. This technique, performed using Refractive Lenticule Extraction (ReLEx®) on the VisuMax® (Carl Zeiss Meditec, Jena, Germany) platform, allows for refractive correction without the need to create a corneal flap. Rather, a small side cut incision, less than 4 mm in size is created at an approximate depth of 80 to 160 μm in the cornea for lenticule extraction. This gives access to the intrastromal pocket created by the preceding lenticule cuts. By creating an intrastromal pocket rather than the traditional flap, surgeons can eliminate associated complications including incomplete and irregular flap cuts, thin flaps, buttonholes, and free caps.

In the past, enhancement following ReLEx® SMILE was a challenge and a number of possible approaches have been considered. Surgeons may contemplate surface ablation if such ablation does not reach the pocket interface. An intraocular lens could be inserted to correct a larger refractive error if the residual stroma is inadequate for photoablation. Other options for the surgeon can either be laser in situ keratomileusis (LASIK), anterior to the SMILE pocket or an additional SMILE procedure, anterior (or posterior, depending on how deep the SMILE pocket is situated) to the initial one. Carl Zeiss Meditec has recently provided an additional option with the development of a series of four circle patterns, programmed within the VisuMax® platform, which can be utilized to create a corneal flap after previous refractive correction with ReLEx® SMILE. This strategy allows for the original SMILE incision pocket to be converted into a LASIK-like flap that can be easily lifted to allow for stromal ablation of the residual refractive error with an excimer laser. Intrastromal incisions include the creation of a lamellar ring, anterior, posterior or adjacent to the previous SMILE pocket cut; a side cut with a hinge and a junction cut from the inner edge of the lamellar ring to junction depths.

In 2013, Riau et al. investigated the use of four different VisuMax® circle patterns when they performed −6.00 D spherical correction using ReLEx® SMILE on six New Zealand white rabbits (12 eyes), and then 28 days later, one of the four circle patterns was used on each of the 12 eyes and evaluated for ease of flap lift [6]. In that animal model, it was determined that pattern D, a lamellar ring adjacent to the cap cut, was the most optimal pattern for flap creation, and ultimately, SMILE re-treatment [8].

Here, we describe the four different VisuMax® circle patterns, which have been programmed to create a corneal flap. We also present the efficacy and safety results for 28 patients upon whom circle pattern D was utilized to create a flap for residual lenticule extraction or refractive enhancement by stromal excimer laser ablation after ReLEx® SMILE. We also discuss the other applications for which the circle patterns can be utilized. To our knowledge, this is the first report of the circle pattern use in human eyes.

## Methods

### Patients

This prospective clinical case study consisted of participants recruited from TRSC International LASIK Center. Each was provided written informed consent that explained the details of the procedure and study protocol in accordance with the principles of the Declaration of Helsinki. According to the Food and Drug Administration (FDA) Thailand, this study was considered clinical quality control and evaluation under CE mark status and therefore, ethical approval was not necessary. In order to be included, patients had to be a minimum of 18 years of age, had previously undergone ReLEx® SMILE and now had a residual refractive error from undercorrection, overcorrection or regression, including residual and/or consecutive astigmatism, that necessitated planned circle pattern treatment and LASIK touch-up. Additionally, a patient with residual lenticule following ReLEx® SMILE, which required extraction was also included. For patients with a residual refractive error, the current refraction had to be stable for at least 3 months. The determination if the previous SMILE incision could be opened, depended on whether one of the two interfaces above or below the lenticule could successfully be separated. Patients had to have the ability to attend postoperative assessment appointments at 1 day, 1 week, 1 month, and 3 months. Patients were excluded if they had ocular conditions, other than residual myopia, consecutive hyperopia with or without residual or consecutive astigmatism following the original ReLEx SMILE procedure.

### Assessments

Preoperatively, all post-ReLEx SMILE patients underwent a complete eye examination, which included objective and manifest refractions, visual acuity (ETDRS LogMAR chart), scotopic pupil size evaluation (Colvard Light-Amplification Pupillometer, Oasis Medical, San Dimas, CA), computerized corneal topography (Orbscan, Bausch & Lomb, Bridgewater, NJ), pachymetry (DGH Technology, Inc., Exton, PA), wavefront analysis (Wasca Analyzer, Wavefront Sciences, Albuquerque, NM), keratometric measurements (Sim-K values from Orbscan), and slit-lamp examination.

The first day after the circle enhancement procedure, patients had their uncorrected visual acuity assessed, a slit-lamp evaluation performed, and any complications or adverse events examined. At each of the remaining postoperative appointments (1 week, 1 month, 3 months) patients were assessed for corrected distance visual acuity, uncorrected visual acuity, objective and manifest refractions, computerized corneal topography, wavefront analysis, keratometric measurements, and slit-lamp examination.

### Surgical technique

All patients had previously undergone ReLEx® SMILE for myopic and/or astigmatism correction at our center and it was determined that further correction would be beneficial. Thus, the circle pattern procedure was performed to create a flap from the original SMILE pocket. In one eye the circle pattern procedure was planned to remove residual lenticule that was present.

**Fig. 1:**
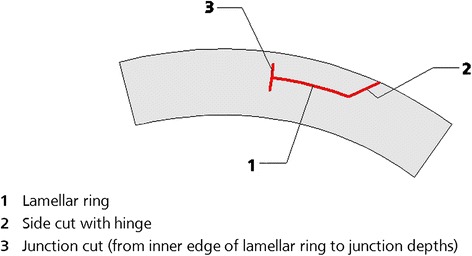
Circle allows for the planning and creation of these corneal cut patterns

**Fig. 2:**
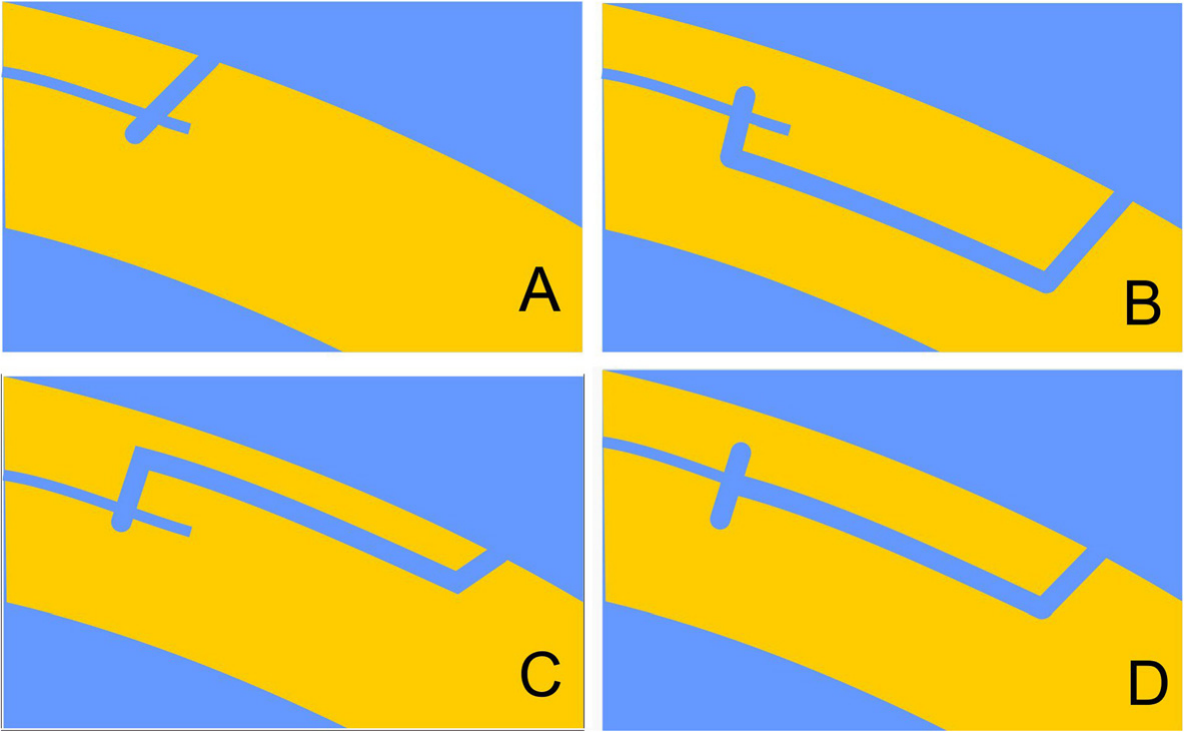
The four different circle patterns. **a.** Direct pocket edge cut without lamellar ring cut, **b.** Lamellar ring cut to enlarge the resulting flap, at a greater depth than the pocket, **c.** Lamellar ring cut to enlarge the resulting flap, at a lesser depth than the pocket, and **d.** Lamellar ring cut to enlarge the resulting flap, at the same depth as the pocket

**Fig. 3:**
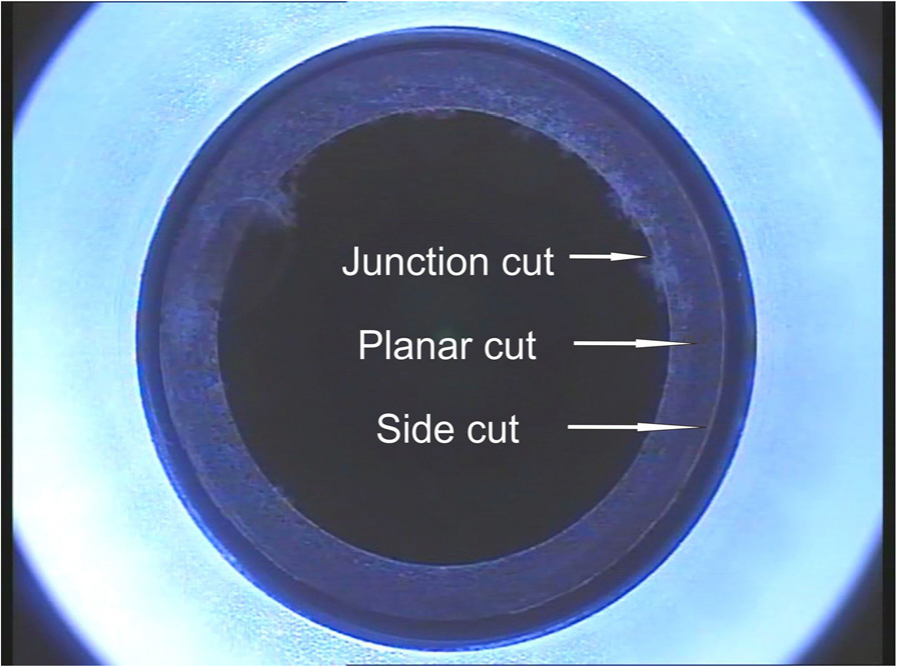
Ring pattern created by the femtosecond laser cuts

Figure 1 illustrates the 3 cut sequences permitted by the circle patterns, which include a lamellar ring, a side cut with hinge and a junction cut that links the inner edge of the lamellar ring to the junction depths. The four different circle patterns (Fig. 2) were composed out of the 3 cut sequences. Pattern A creates a side cut within the boundaries of the SMILE pocket cut. The side cut borders on the outer diameter of the lamellar cut that extends to the surface of the cornea and the angle of the side cut impacts the diameter of the side cut on the surface; pattern B creates a lamellar ring, posterior to the SMILE pocket cut; pattern C creates a lamellar ring, anterior to the SMILE pocket cut; and pattern D creates a lamellar ring adjacent to the SMILE pocket cut, at the same depth of the pocket. All eyes treated in this series were treated using pattern D. Patterns B, C, and D resulted in a ring pattern (Fig. 3).

**Fig. 4:**
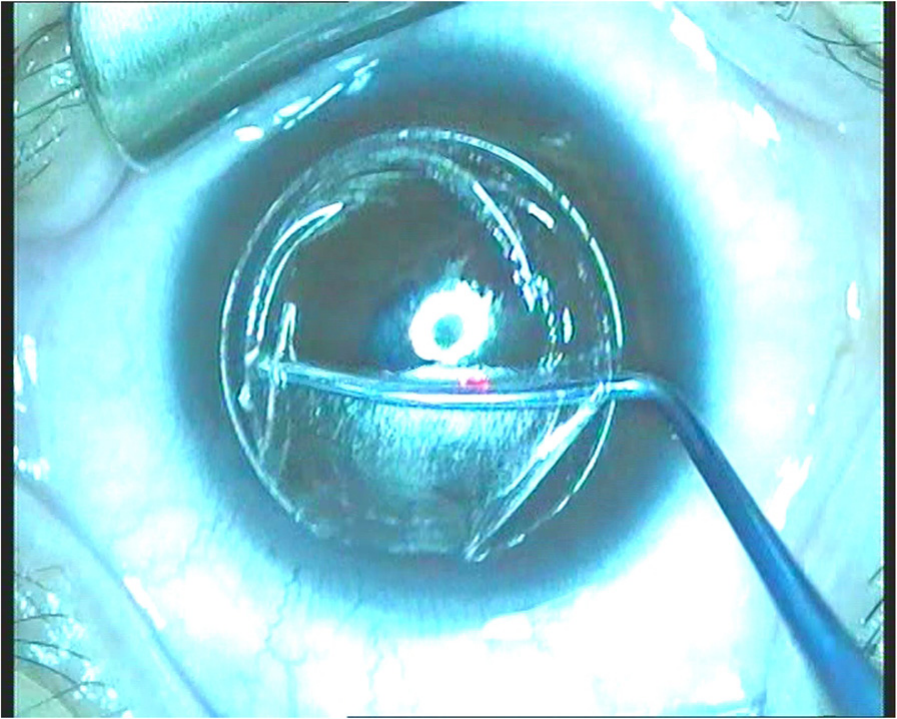
The Chansue ReLEx® Dissector (CRD) was used to separate and lift the flap

Prior to commencing the circle procedure, preservative-free anesthetic (Tetracaine Hydrochloride 0.5 %, Alcon Corporation, Switzerland) was administered into the patient’s conjunctival sac. The eye underwent standard sterile draping, and insertion of the lid speculum. The surgical microscope was adjusted to a magnification of 0.6x. The patient’s eye was centered and docked with the curved interface cone before application of suction fixation. Specific attention was paid to centering the circle treatment to the previously performed SMILE cuts in order to ensure that the new lamellar ring meets the existing cap in all areas. The laser treatment was automatically based on the selected pattern. The VisuMax laser settings for the 27 eyes included small size cone, energy level of 160 joules, 7.9 mm lamellar diameter with junction diameter of 6.5 mm, depth of 80 to 140 μm (according to the previous SMILE pocket depth), side cut angle at 90 degrees while the additional eye (removal of residual lenticule) had the same settings with exception of 8.0 mm lamellar diameter. The original SMILE procedure pocket diameter setting was 7.5 mm with depths ranging from 80 to 140 μm. Once the laser was complete and suction was released, ultrasonic pachymetry was performed in the central cornea. A Sinsky hook was used to separate the edge of the newly created flap near the hinge. A Chansue ReLEx® Dissector (CRD) was then used to carefully separate the flap bed from any remaining lamellar ring adhesions and the flap was gently lifted (Fig. 4). Ultrasonic pachymetry was then repeated on the stromal bed. The central flap thickness was calculated by subtracting the second pachymetry from the first. For eyes requiring refractive correction, following creation of the flap, patients underwent enhancement correction by stromal ablation with the Carl Zeiss MEL80 excimer laser, typically at the ablation diameter of 6.0 mm plus 1.0 mm of transition zone. For the one eye, the lenticule residue was removed manually.

### Outcome measures

The primary endpoint of this series was to evaluate the ability of the surgeon to create a flap using the circle pattern and successfully lift this flap to perform refractive enhancement to the original SMILE procedure. In enhancement cases, the stromal bed should be of sufficient quality to perform LASIK. Safety of the use of the circle pattern was also evaluated, and assessments of safety included the loss of 2 or more Snellen lines of CDVA [7] after 3 months, compared to preoperative assessment, and any procedural or flap complications including rupture or perforation.

### Statistical analysis

All patient demographic and baseline information as well as outcome measurement data were collected on paper case report forms, which subsequently were entered into an Excel spreadsheet (Microsoft Corporation, Redmont, WA). Descriptive analyses were performed using the data analysis features of Excel. Analysis of visual acuity results were performed by calculating the geometric mean with standard deviation into logMAR format from Snellen examination results.

## Results

**Fig. 5:**
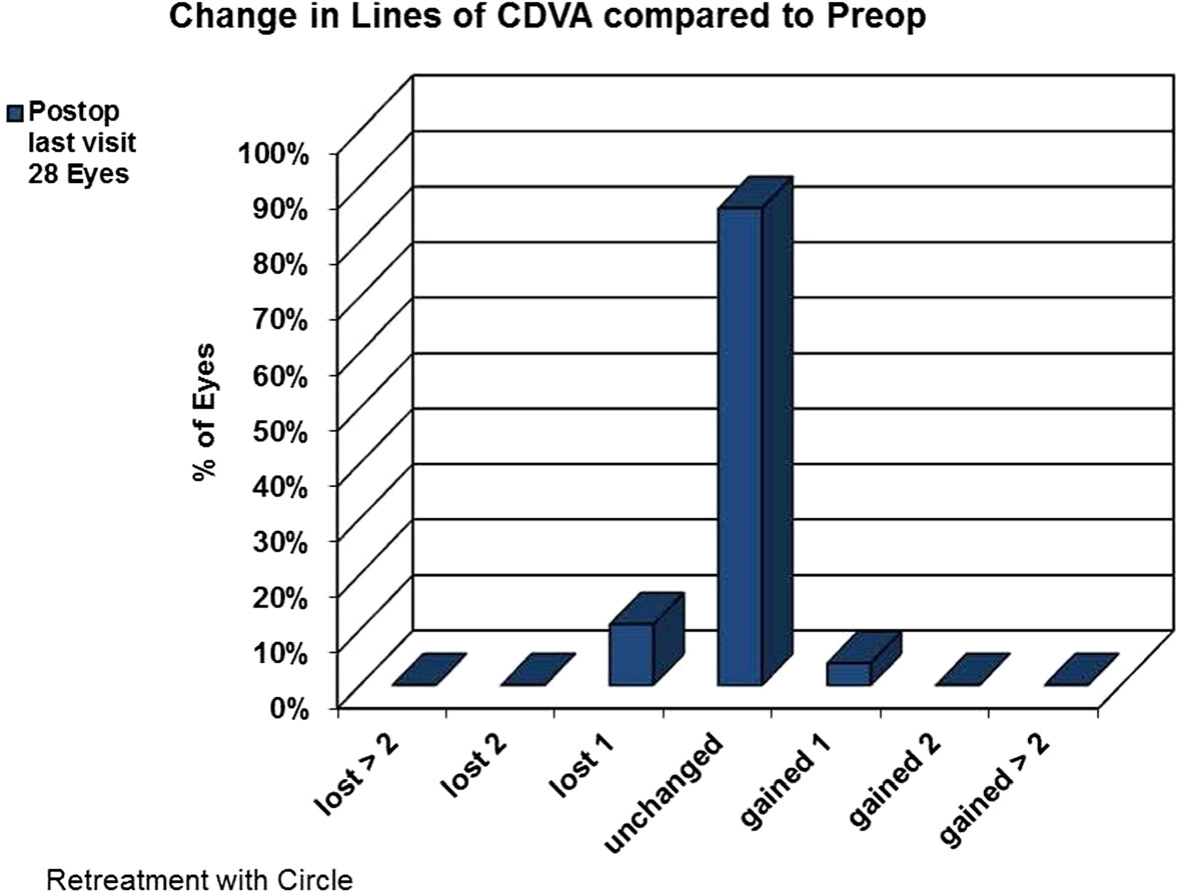
Percentage of eyes having a change in lines of CDVA at 3 months versus preoperatively

Twenty-eight eyes were enrolled in the study and underwent flap creation using the circle pattern D. Twenty-seven eyes had flap creation for refractive enhancement with an excimer laser and one eye was treated with circle for the removal of residual lenticule. The average duration between the original SMILE surgery and CIRCLE is 202 (SD = 95, min = 69, max = 406) days. Table 1 shows the preoperative demographics of the study population. In 100 % of cases (28 eyes) the lift of the flap was successfully created as planned. In all cases of refractive enhancement (27 eyes) by LASIK, the quality of the stromal bed was deemed sufficient in smoothness for subsequent excimer laser ablation. The average flap thickness was 116 (SD = 18.8, min = 73, max = 153) μm. Of the 28 eyes that underwent the circle pattern D for flap creation, none lost two or more lines of CDVA after 3 months. Figure 5 shows the loss and gain of CDVA lines. No flap-related complications (rupture, perforation, miscreation) occurred, and no circle related complications (debris, tissue in the interface) occurred in any eye.

**Table 1:** Preoperative demographics of the study population

Parameter		
Eyes	28 (12 OD/16 OS)	
Male/Female	46 % / 54 %	
	Mean ± SD	Range
Age (mean)	36 ± 11 years	19 to 57 years
Manifest Spherical Equivalent (D)	−0.74 ± 0.80	0.375 to −2.875
Manifest Cylinder (D)	−0.7 ± 0.34	0 to −1.25

In one eye in this series, the femtosecond laser was incorrectly programmed, and the plane was cut at an incorrect depth, necessitating the use of micro-scissors to connect the planes. This was performed uneventfully, and healing process and visual results are similar to those of the other cases. There were no serious adverse events or serious side effects reported.

At three months post-operatively, uncorrected visual acuity was 20/40 or better in 100 % of the eyes, and 20/20 or better in 95.8 % of the 24 eyes that were corrected for distance vision. Residual refractive error (spherical equivalent) averaged +0.1 D.

## Discussion

ReLEx® SMILE has been used clinically over the past few years to provide patients with refractive correction by means of lenticule extraction [8]. By leaving the cornea intact, surgeons are able to maintain its biomechanical stability, while protecting more of the nerve fibers. Studies have demonstrated the safety, predictability and efficacy of SMILE in the correction of myopia [1–5, 7, 9]. In a recent study by Hjortdal et al., they found that 3 months after patients were treated with SMILE, 94 % were within ±1.00 D [3]. Similarly, Sekundo and colleagues found that 95.6 % of eyes in their cohort were within ±1.00 D [2]. Reported predictability of LASIK ranges widely from 78.2 % up to 96.7 % [10, 11] with enhancement procedures typically being performed with flap lift LASIK or surface ablation [12, 13]. The higher rates of predictability anticipated with SMILE suggest that enhancement rates will be lower than with LASIK. Because SMILE does not cut a full circumferential flap there is improved maintenance of corneal integrity, and refractive regression is not anticipated to be as common as it is in LASIK. Studies have also shown SMILE procedures deliver stable results with minimal, non-significant refractive regression up to 6 months postoperatively [1–5, 8]. The good predictability, stability and efficacy results suggest that SMILE is a viable choice for refractive correction. Previously, for the few patients that did require enhancement, options were limited to an implantable lens or excimer laser surface ablation (PRK).

In our series, we present the option of using the circle-created flaps for eyes previously treated with SMILE. Riau et al., who first evaluated the use of the circle patterns in rabbit eyes, determined that pattern D, a lamellar ring adjacent to the cap cut, was the most optimal pattern for flap creation and ultimately SMILE re-treatment [6]. In our center, pattern D was also the pattern of choice because choosing to create a flap at the same depth as the original cut seemed to be the most appropriate in all cases.

In all of our patients, lifting the circle-created flap through the use of pattern D could be easily achieved, similar to lifting a femtosecond LASIK flap. The study by Riau et al. also evaluated the ease of flap lift, comparing the four different circle patterns on rabbit eyes. They determined that patterns A and D were the easiest to lift, and the resulting flap bed remained smooth and undisrupted [6].

Of interest was the thickness of the created flaps. As the authors routinely performed intraoperative direct ultrasonic pachymetry on the stromal bed before excimer laser ablation (to ensure adequate thickness after the ablation) in enhancement procedures, the thickness of each flap was also obtained by subtracting the thickness of the stroma bed from the total corneal thickness, also taken with intraoperative, direct ultrasonic pachymetry. (Alternatively, SMILE cap thickness can be measured with Anterior Segment OCT [14]. Table 2 compares the thickness of the flaps calculated in this fashion to the cap thickness (depth) settings of the femtosecond laser in the original ReLEx SMILE surgery. The discrepancy can be partially explained by the changes in the epithelial thickness as a result of epithelial remodeling after the original surgery, which may be related to the amount of myopic correction [15].

**Table 2:** Flap thickness compared to settings in previous ReLEx SMILE surgeries

Eye No.	Flap thickness (μm)	Cap thickness setting in the previous SMILE procedure (μm)
1	111	100
2	126	120
3	73	80
4	115	100
5	146	140
6	138	140
7	104	100
8	153	140
9	114	100
10	116	100
11	123	120
12	114	100
13	84	80
14	120	120
15	135	140
16	132	140
17	125	100
18	143	140
19	118	120
20	113	100
21	122	120
22	86	80
23	90	100
24	106	100
25	109	100
26	113	100
27	106	100
28	125	120

As this is the initial report of the clinical use of circle pattern D on human eyes following SMILE, we acknowledge some limitations. Here we only looked at the technical efficacy of the procedure for these patients and the presence of any safety concerns. We intend to further investigate and analyze other variables, such as OCT data, confocal microscopy and patient satisfaction, to provide additional insight into this procedure. We feel that further study needs to be conducted to determine if the time period between the initial SMILE procedure and the Circle procedure influences the ease of flap lift.

## Conclusion

Although this initial series only followed 28 eyes for a period of 3 months, we feel that it provides useful initial information and demonstrates that the VisuMax® circle pattern D is technically feasible and is a suitable method to create a corneal flap for enhancement following SMILE.
